# Long-Acting Paliperidone Parenteral Formulations Based on Polycaprolactone Nanoparticles; the Influence of Stabilizer and Chitosan on In Vitro Release, Protein Adsorption, and Cytotoxicity

**DOI:** 10.3390/pharmaceutics12020160

**Published:** 2020-02-16

**Authors:** Mohammed Elmowafy, Nabil K. Alruwaili, Khaled Shalaby, Khalid S. Alharbi, Waleed M. Altowayan, Naveed Ahmad, Ameeduzzafar Zafar, Mohammed Elkomy

**Affiliations:** 1 Department of Pharmaceutics, College of Pharmacy, Jouf University, Sakakah P.O. Box 2014, Saudi Arabia; nkalruwaili@yahoo.com (N.K.A.); khsalalbym@yahoo.com (K.S.); naveedahmd@yahoo.com (N.A.); Zafar123@yahoo.com (A.Z.); komy1988@yahoo.com (M.E.); 2 Department of Pharmaceutics and Industrial Pharmacy, Faculty of Pharmacy (Boys), Al-Azhar University, 11751 Nasr City, Cairo, Egypt; 3 Department of Pharmacology, College of Pharmacy, Jouf University, Sakakah P.O. Box 2014, Saudi Arabia; khalaharbi@yahoo.com; 4 Pharmacy Practice Department, College of Pharmacy, Qassim University, Qassim 51452, Saudi Arabia; waleedmalt@hotmail.com; 5 Department of Pharmaceutics and Industrial Pharmacy, Faculty of Pharmacy, Beni-Suef University, 62521 Beni-Suef, Egypt

**Keywords:** paliperidone, polycaprolactone nanoparticles, controlled release, stabilizer, chitosan coating, cytotoxicity

## Abstract

Long-acting preparations containing the antipsychotic paliperidone for intramuscular injection has drawn considerable attention to achieve harmless long-term treatment. This study aimed to develop paliperidone loaded polycaprolactone (PCL) nanoparticles and investigate the influence of PCL/drug ratio, stabilizer type, and chitosan coating on physicochemical properties, protein adsorption, and cellular toxicity. Results showed that chitosan coating produced enlarged particle sizes, shifted the surface charges from negative into positive and did not influence encapsulation efficiencies. Chitosan coating relatively sustained the drug release especially in pluronic stabilized formulations. Pluronic F127 based formulations exhibited the least protein adsorption (384.3 μg/mL). Chitosan coating of Tween 80 and polyvinyl alcohol stabilized formulations significantly (*p* < 0.05) increased protein adsorption. Cellular viability was concentration-dependent and negatively affected by stabilizers. All formulations did not show cellular death at 1.56 μg/mL. Inflammatory responses and oxidative stress were less affected by Tween 80 compared with other stabilizers. Chitosan minimized all aspects of cellular toxicity. Collectively, stabilizer type and chitosan coating play critical roles in developing safe and effective long-acting PCL nanoparticles intended for parenteral drug delivery. The coated formulations containing Tween 80 and Pluronic F127 as stabilizers are warranted a future in vivo study to delineate its safety and efficacy profiles.

## 1. Introduction

**Figure 1:**
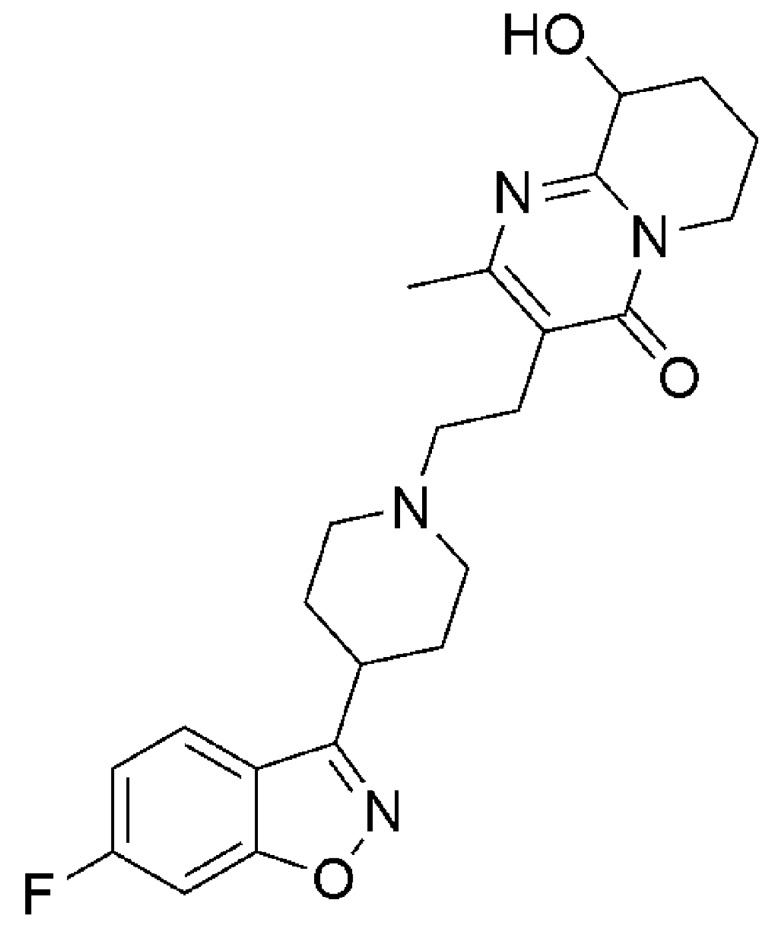
Chemical structure of paliperidone.

Paliperidone (PP) is a benzoxazole derivative clinically used in the treatment of psychosis and schizophrenia (Figure 1). The mechanism of action includes dopaminergic, serotonin, adrenergic, and histamine receptors’ antagonism. It is the major active metabolite of the atypical antipsychotic risperidone after extensive liver metabolism via hydroxylation (PP is 9-hydroxy-risperidone) [1].

Long-acting preparations containing PP for intramuscular injection have drawn considerable attention due to their potential to achieve harmless long term treatment. Commercially available PP palmitate suspension (Invenga^®^ Sustennatm and Xeplion^®^, Janssen Pharmaceutica NV, Beerse, Belgium) is slowly hydrolyzed into active PP producing prolonged antipsychotic effect. This approach is associated with problems such as individual variations in metabolic capacity, sudden failure in drug release into systemic circulation and undesired subclinical inflammatory reaction. Large amounts of crystalline PP have been reported to accumulate in infiltrating macrophages leading to neovascularization after Xeplion^®^ administration [2].

Polymeric devices/drug delivery systems have been proposed to prolong the antipsychotic effect of PP. Wang et al. developed a poly(d,l-lactide-*co*-glycolide) (PLGA)/dimethyl sulfoxide in situ forming implant system capable of achieving more than a 3-week sustained release and antipsychotic activity in vivo [3]. Recently, Nanaki et al. designed a high surface area mesoporous silica foam incorporated into polymeric microparticles (PLA and PLGA) to enhance PP solubility and prolong its action for 10 to 15 days [4].

Nanoparticle-based delivery systems have got a lot of consideration nowadays due to their unique characteristics which include non-immunogenicity, stability in blood, non-toxicity, non-inflammatory and non-activation of neutrophils [5]. Basically, hydrophobic drugs can be delivered by different nanoparticulate systems such as lipid-based nanoparticles [6], polymeric nanoparticles [7] and lipomer (polymeric lipid hybrid nanoparticles) [8]. Noteworthy, polymeric nanoparticles (PN) possess a wide range of applications in tissue engineering and drug delivery. Especially for biodegradable and biocompatible types, they can improve bioavailability and safety, control the release of encapsulated drugs [9] as well as overcoming physiological barriers [10]. PCL is a semi-crystalline polyester that is FDA approved as a biodegradable and biocompatible polymer. Moreover, PCL nanoparticles can entrap both hydrophilic and lipophilic drugs for controlled release [11], provide a stable system in biological fluids and introduce low toxicity in vitro and in vivo [12].

Preparing PN is a complex process that commonly requires formulating with nanoparticle stabilizers. A variety of different stabilizers have been exploited up till now with PCL nanoparticles. These stabilizers include nonionic surfactants such as Tween 80 [13] and polymeric stabilizers such as Pluronic F68 [14], Pluronic F127 [15], and polyvinyl alcohol [16]. Such excipients are expected to influence product stability, release behavior, and safety. One way to modulate the previously mentioned attributes is to coat the PN with hydrophilic polymers. Chitosan is a good candidate owing to its excellent biocompatibility and biodegradability that has been behind its widespread use in the fields of medicine, agriculture, and pharmaceutical, food, and chemicals industry.

Although PCL nanoparticles seem to be one of the most relevant carriers for safe and controlled delivery of drugs, it has never been exploited as a potential carrier for PP. Accordingly, the aim of this work was to formulate PP loaded PCL nanoparticles and investigate the influence of PCL/drug ratio, stabilizer type, and high molecular weight chitosan coating on their physicochemical properties, stability, protein adsorption and cytotoxicity. Our ultimate goal was to develop safe and effective long-acting PCL nanoparticles intended for parenteral delivery of PP.

## 2. Materials and Methods

### 2.1. Materials

PP was purchased from Boss Chemical Industry Co., Ltd. (Jinan, China). PCL (Mw: 70,000–90,000 Da), Pluronic F68, Pluronic F127, polyvinyl alcohol (PVA; Mw: 15,000–20,000) and Tween 80 were purchased from Sigma Aldrich (St. Louis, MO, USA). Chitosan (Mw: 100,000–300,000 Da) was purchased from Acros organics (Geel, Belgium). Bovine serum albumin (BSA) was purchased from Techno Pharmchem (New Delhi, India). The reagents RPMI-1640 medium, 3-(4,5-dimethylthiazol-2-yl)-2,5-diphenyltetrazolium bromide (MTT) and dimethylsulfoxide (DMSO) were purchased from Sigma (St. Louis, MO, USA). Fetal Bovine serum was purchased from Gibco (Loughborough, UK). Other chemicals, reagents, and solvents used were of analytical reagent grade. Highly purified water (Smart2Pure, Thermo Scientific, Stockholm, Sweden) was used for the preparation of all buffer and water-based solutions.

### 2.2. Preparation of PP Loaded PCL Nanoparticles

PP loaded PCL nanoparticles were prepared by the nanoprecipitation method reported by Fessi, et al. [17] with some modifications. During development, we evaluated the ratios of PP: PCL (1:2 and 1:4 *w*/*w*) with a fixed ratio of the organic phase to the aqueous phase (1:2 *v*/*v*). The organic phase consisted of acetone/ethyl acetate mixture (1:1 *v*/*v*) in which appropriate amounts of PP and PCL were dissolved under mild heating (45 °C in tightly closed containers) and magnetic stirring (200 rpm) till a clear solution is obtained. To provide the aqueous phase, 0.5% of different stabilizers (Tween 80, Pluronic F68, Pluronic F127 and PVA) were dissolved in purified water with the aid of magnetic stirring. Preliminary dispersion in heated water was necessary in the case of PVA. Thereafter, the organic phase was injected dropwise into the aqueous phase under magnetic stirring at 600 rpm. Stirring was continued for 4 h until complete evaporation of the organic solvent. The resultant suspension was filtered (0.45 μm) to obtain a suitable nanosized range. The filtrate was subjected to ultracentrifugation (16,000 rpm) at 10 °C for 30 min and the supernatant containing unentrapped PP was separated.

For coated formulations, nanoparticles’ precipitate was dispersed in 1 mL 0.5% (*w*/*v*) chitosan solution, prepared in 1% acetic acid, and then placed in a shaking incubator (Julabo SW22 GmbH, Seelbach, Germany) at 150 rpm for 4 h at room temperature. After incubation, the nanoparticles were recovered by ultracentrifugation (16,000 rpm, 10 °C for 30 min) and then resuspended in purified water. The resulting supernatant was analyzed to quantify unentrapped PP. The composition of different formulations is outlined in Table 1.

### 2.3. Particle size and Zeta Potential

Particle size (nm), polydispersity index (PI) and zeta potential of PP loaded PN formulations were determined in a Malvern particle size analyzer (Zetasizer Nano ZS, Malvern Instruments, Worcestershire, UK). Formulations were diluted with purified water, in a ratio of 1:10 (*v*/*v*) at room temperature before measurement. All measurements were done in triplicates.

### 2.4. Production Yield and pH

Production yield was considered as the practical yield of dried nanoparticles (after filtration with 0.45 µm filter) recovered from each batch with respect to the total amount of drug and polymers used in each batch. The percentage yield was computed using the following equation:

$$\mathrm{Yield}\;\%=\frac{\mathrm{Practical}\;\mathrm{mass}\;\mathrm{of}\;\mathrm{nanoparticles}}{\mathrm{total}\;\mathrm{added}\;\mathrm{amount}\;\mathrm{of}\;\mathrm{PP}\;\mathrm{and}\;\mathrm{PCL}}\times 100$$

For pH determination, samples were measured by a pH meter (HI 2211 pH/ORP meter, HANNA instruments, Woonsocket, RI, USA) without dilution.

### 2.5. PP Encapsulation Efficiency (EE)

EE% was determined by measuring entrapped PP in nanoparticles in each batch (direct method). After nanoparticles separation by centrifugation, nanoparticle deposited pellets were dissolved in acetone/ethyl acetate mixture (1:1, *v*/*v*), vortexed (DragonLab, Thermo Scientific, Pudong New Area Shanghai, China) for 5 min and then analyzed at 323 nm in a UV–Visible spectrophotometer (Genesys 10S UV-VIS, Thermo Scientific, Pudong New Area Shanghai, China). The calibration curve for the UV assay of PP was constructed using five concentrations ranging from 2.5–25 μg/mL. The correlation coefficient (*r*^2^) was about 0.997. EE% of PP was calculated according to the following equation:

$$\mathrm{EE}\%=\frac{\mathrm{Amount}\;\mathrm{of}\;\mathrm{PP}\;\mathrm{analyzed}}{\mathrm{Total}\;\mathrm{added}\;\mathrm{amount}\;\mathrm{of}\;\mathrm{PP}}\times 100$$

### 2.6. In Vitro Release of PP from PN

In vitro release studies were carried out by a dialyzing/diffusion technique using a screw-capped Spectra/Por^®^ dialyzing tube (Float-A-Lyzer^®^ G2, with Biotech Cellulose Ester Membrane, 8000–10,000 Da MWCO, Sigma Aldrich, St. Louis, MO, USA) tightly closed at both ends. Tubes were previously soaked in purified water for 30 min. An aliquot of 1 mL of PN (equivalent to 10 mg PP) was put inside the dialyzing tube which was immersed in tightly covered (to prevent evaporation of release medium) glass beaker containing 400 mL phosphate-buffered saline (PBS) of pH-7.4 to maintain sink conditions (the solubility of PP in receptor media was about 0.134 mg/mL). The whole set was placed on a magnetic stirrer adjusted at 37 ± 2 °C and 100 rpm. At appropriate time points (1, 2, 3, 4, 6, 8, 10, 12, 14, 16, 20, 24, and 28 days), 2 mL aliquot was withdrawn from the release medium and the same volume of fresh medium was added back to maintain a constant volume. Withdrawn samples were diluted with acetone/ethyl acetate mixture (1:1, *v*/*v*) and the percentage of PP released was determined spectrophotometrically at 323 nm. All samples were measured in triplicate. The cumulative amount of PP released from different formulations after 28 days was statistically compared.

### 2.7. Stability

Shelf stability of the investigated formulations was assessed by storing PN in sealed glass vials at room temperature for 3 months. Investigated parameters included particle size, zeta potential, pH and EE%. All measurements were carried out in triplicates.

### 2.8. Thermal Study

Differential scanning calorimetry (DSC) was used to study potential interaction between PP and PN components through analysis of phase transition temperature. Accurately weighed 5 mg of PP, PCL, stabilizers, physical mixtures and F3 and F4 (as examples of uncoated and coated formulations respectively) were placed in aluminum pans (140 µL capacity), crimped and analyzed by Mettler DSC (DSC3, Mettler Toledo, Switzerland). A standard empty aluminum pan was used as a reference. Thermograms were recorded between 30 °C and 275 °C at a scan rate of 10 °C/min under nitrogen gas. Data were evaluated with STARe 15.00 software.

### 2.9. Fourier Transform-Infrared Spectroscopy

Interaction between PP and PN components was further verified by Fourier transform infrared spectroscopy (FT-IR). FT-IR analysis was carried out between 4400 and 350 cm^−1^ in transmission mode for PP, PCL, Pluronic F127, chitosan, F3 and F4 (as examples of uncoated and coated formulations respectively).

### 2.10. Morphology

The shape and appearance of PN were evaluated by Scanning Electron Microscopy (SEM, JOEL, Akishima, Tokyo, Japan). F1, F3 and F7 (as examples of uncoated formulations) and F2, F4, and F8 (as examples of corresponding coated formulations) were diluted with double distilled water (100 folds dilution) and dropped on a flat metallic sample holder and allowed to dry overnight at room ambient temperature. Samples were coated with a gold thin layer using a gold sputter unit. After that, samples were photographed at an acceleration voltage of 10 kv.

### 2.11. Protein Adsorption

BSA was used to assess protein adsorption onto nanoparticles’ surface by the method described by Kim, et al. [18] with slight modification. One milliliter of BSA solution (1% in PBS; pH = 7.4) was mixed with 0.5 mL of the investigated formulations and incubated at 37 °C for 48 h. Non-adsorbed BSA was removed in the supernatant by centrifugation at 15,000 rpm for 15 min. Nanoparticle’s pellets were washed thrice with PBS and 250 μL of Biuret reagent was added. Biuret reagent/BSA-complex was then determined spectrophotometrically at 540 nm [19]. A standard curve was previously constructed with known concentrations of BSA.

### 2.12. Cytotoxic Effects

#### 2.12.1. Cell Viability

Formulations were assessed for cytotoxicity using the human lung fibroblast cell line (WI-38; as normal cell model) by MTT assay. The cell line was obtained from ATCC via a holding company for biological products and vaccines (VACSERA, Cairo, Egypt). The cells were cultured in RPMI-1640 medium (pH = 7.4) with 10% fetal bovine serum. Antibiotics added were 100 units/mL penicillin and 100 µg/mL streptomycin. The cells were seeded in a 96-well plate at a density of 1.0 × 10^4^ cells/well at 37 °C in a 5% CO_2_ incubator for 48 h. To study the effect of concentration, the cells were treated with different concentrations of different formulations after dilution with culture media and then incubated for 48 h. After incubation, 20 µL of MTT solution at 5 mg/mL was added and incubated for 4 h. After that, 100 µL of DMSO was added to dissolve the purple formazan formed. The colorimetric assay was performed at an absorbance of 570 nm using a plate reader (EXL 800, ChroMate-4300, Palm City, FL, USA). This colorimetric assay is based on the conversion of the yellow tetrazolium bromide to a purple formazan derivative by mitochondrial succinate dehydrogenase in viable cells. Experiments were performed using culture media as negative control and stabilizer free formulation (SFF) as a positive control. SFF was prepared by the same above mentioned procedure with sonication (4710 series-Crest Ultrasonic Corp., San Diego, CA, USA) for 10 min at 50 W in an ice jacket. Based on the results of cell viability, nanoparticles concentration 50 μg/mL was used for the next two experiments.

#### 2.12.2. Cytokine Secretions

Cytokine (tumor necrosis factor-alpha (TNF-α) and interleukin-6 (IL-6)) secretion following incubation with nanoparticles was assessed using the enzyme-linked immune-sorbent assay technology (ELISA) according to manufacturer standard procedure. Cells were incubated with different formulations for 24 h and clear supernatants were obtained by centrifugation at 2000× *g* for 20 min at 4 °C to remove insoluble impurities and cellular debris. The diluted supernatants (0.1 mL) were added into an anti-cytokine pre-coated 96-well plate which was covered and incubated at 37 °C for 90 min. Then 0.1 mL of biotin-conjugated antibody and 0.1 mL of streptavidin horseradish peroxidase (HRP) were added. Tetramethyl benzidine (TMB) substrates were then added to visualize HPR enzymatic reaction. The density of resultant yellow color was measured at 450 nm and the concentration (Pcg/mL) was calculated from reference standard curves. All measurements were done in triplicates.

#### 2.12.3. Oxidative Stress

After collecting cells by centrifugation (as mentioned in the above section), cells were homogenized in cold buffer (50 mM potassium phosphate, pH 7.5, 2 mM EDTA). Then the homogenates were centrifuged at 4000 rpm for 15 min at 4 °C and supernatants were removed for the assay of malondialdehyde (MDA) and reduced glutathione (GSH). In the case of MDA assay, 0.2 mL of homogenates were mixed with 1 mL of thiobarbituric acid (TBA) and left for 30 min at 95 °C. The resultant pink product was measured at 534 nm. In the case of GSH assay, 0.1 mL of homogenates were mixed with 0.5 mL of trichloroacetic acid (TCA) and left for 5 min at room temperature and then centrifuged at 3000 rpm. Aliquots of 0.5 mL were mixed with 0.1 mL 5, 5’ (dithiobis (2-nitrobenzoic acid) (DTNB) and left for 10 min. The resultant yellow product was measured at 405 nm. All measurements were done in triplicates.

### 2.13. Statistical Analysis

All results were analyzed by one-way ANOVA and means were compared by Tukey’s multiple comparison testing using GraphPad Prism V. 5 software. The inhibitory concentration of 50% of cells (IC50) was calculated by nonlinear regression utilizing “dose response-inhibition” equations using the same software. A difference with *p* < 0.05 was considered to be significant.

## 3. Results and Discussion

### 3.1. Preparation of Formulations

PP loaded polymeric nanoparticles were prepared by the nanoprecipitation method. This technique involves dissolving PP and PCL in an organic solvent while dissolving a stabilizer in the aqueous phase. By the addition of the organic phase to the aqueous phase, the organic solvent diffuses into the aqueous medium causing the polymer to precipitate into nanosized particles. Simultaneously, the stabilizer self-assembles around the polymeric nanoparticles directing its hydrophobic tail to the hydrophobic polymer core and hydrophilic head group towards the external aqueous environment. Using ethyl acetate as the organic solvent confers a couple of advantages; (1) ethyl acetate is able to dissolve both the drug and the polymer, (2) ethyl acetate can produce relatively small particles owing to its low interfacial tension and high diffusivity into the aqueous phase enabling efficient polymer dispersion [20]. In this study, three factors; chitosan coating, drug/polymer ratio and stabilizer type were studied and their influence on physicochemical characteristics, drug release pattern and cytotoxicity were investigated.

### 3.2. Particle Size

Particle size is considered a versatile factor that influences drug release and in vivo interaction. Smaller particles have a larger surface area and consequent larger burst effect [9], whereas larger particles have a smaller surface area available for water penetration and diffusion and then provide slower drug release patterns [21]. On the other hand, larger particles can interact more prominently with macrophages and get cleared more rapidly.

Table 2 shows the particle size of different formulations. Tween 80, Pluronic F127 and Pluronic F68 produced nanosized particles (169.1 ± 5.4 nm, 160.8 ± 3.7 nm, and 152.4 ± 4.7 nm, respectively) with homogenous and uniform particle size distribution as evidenced by PDI values of 0.116 ± 0.051, 0.073 ± 0.058 and 0.076 ± 0.058, respectively. Using PVA produced significantly (*p* < 0.05) larger particle size (360.3 ± 28.5 nm). A possible explanation for this behavior is that PVA could not be homogenously dispersed/dissolved in the cold aqueous phase, which mandated raising the temperature to 45 °C. The increasing temperature might have led to the dissociation of PVA molecules from nanoparticle surface and migration to the bulk medium leaving the nanoparticles uncoated. This, in turn, could cause particle aggregation and consequent sedimentation.

Drug/polymer ratio did not affect particle size significantly (*p* > 0.05) (Table 2) although previous studies reported that the higher the polymer concentration, the larger the particles produced [22,23]. On the other side, chitosan-coated formulations were associated with significant (*p* < 0.05) particle size increase when compared with the corresponding uncoated ones. This might be attributed to the fact that coating was performed after nanoparticle preparation hence chitosan was located onto particles’ surface contributing particle size enlargement.

### 3.3. Zeta Potential

Zeta potential measures the electrokinetic potential of particle surface providing a realistic value of particle surface charge in colloidal systems. Zeta potential can predict stability as the higher the surface charge, the higher the interpaticulate repulsive forces and hence the higher the stability. Table 2 shows zeta potential values of different formulations. In uncoated formulations, particles acquired negative values ranging from −4.57 ± 0.63 mV (F7) to −15.53 ± 0.28 mV (F13). The negative value is ascribed to ionized polyester’s carboxylic terminals [24]. It could also be noticed that the 1:4 drug polymer ratio produced more negative values compared with the 1:2 ratio which is attributed to more exposed carboxyl terminals in the 1:4 ratio. However, the zeta potential of chitosan-coated formulations shifted from negative to positive values owing to the presence of amino groups (–NH3^+^) in chitosan molecules confirming successful particle coating.

### 3.4. Yield%, pH, and EE%

In the determination of the yield %, a fraction of particles below 450 nm was separated to investigate the capability of each stabilizer to achieve the particles in the desired size. Table 2 shows the yield % and EE% of different formulations. Production Yield was found to be in the range between 79.4% and 32.1%. Pluronic F127 (79.4% ± 5.8%) produced the highest, while PVA produced the lowest yield (32.1% ± 6.9%). These values can be explained by the size of particles produced by each of the two stabilizers (Table 2) and confirm the weak surface activity of PVA in PCL polymeric nanoparticles. As expected, the yield percent of chitosan-coated formulations were less than that of the corresponding uncoated ones as chitosan significantly increases particle size.

Unlike yield percentage, pH was not significantly influenced by the type of stabilizer used (Table 2). However, chitosan-coated formulations were of lower pH (range: 5.2 ± 0.24–5.7 ± 0.73) compared with the corresponding uncoated ones (range: 6.1 ± 0.92–6.6 ± 0.28) (Table 2). The presence of acetic acid traces that cause ionization of chitosan amino groups may be responsible for the slight acidic behavior of the chitosan-coated formulations.

Entrapment efficiencies of various formulations were found to be in the range of 51.6 ± 3.3–70.6 ± 6.8%. Satisfactory percentages were obtained by all investigated stabilizers except PVA. As PP is a hydrophobic drug (log *p*~1.8), it is mostly incorporated into the hydrophobic core of the PCL nanoparticle. Accordingly, the coating of nanoparticles with chitosan did not significantly influence the entrapment of PP.

### 3.5. In Vitro PP Release Studies

**Figure 2:**
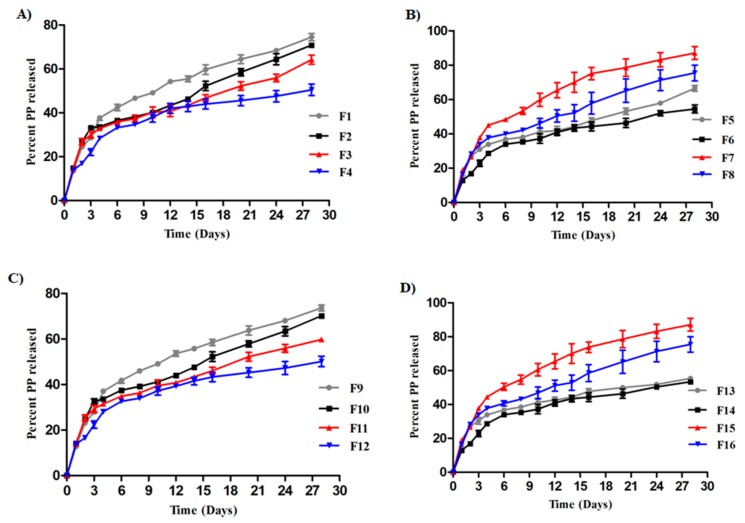
In vitro release profiles of PP from (**A**) F1–F4, (**B**) F5–F8, (**C**) F9–F12, and (**D**) F13–F16 (mean values ± SD, *n* = 3).

The in vitro release pattern of PP from different investigated formulations is depicted in Figure 2. The formulations exhibited biphasic drug release patterns with burst release through the first 4 days followed by sustained release afterward. The fast initial release of PP is probably attributed to untrapped drug bound to the nanoparticle surface. A maximum burst effect was observed in F7 (nearly 45% of the drug was released) whereas F12 showed minimum initial burst release (nearly 28% of the drug was released). The sustained release behavior in the later stages is attributed to the slow diffusion of the hydrophobic drug across the polymeric matrix. PCL is characterized by low water permeability [5] and a very slow degradation rate (2–3 years).

Figure 2 shows that chitosan-coated formulations produced slower release in both stages compared with the corresponding uncoated ones. This observation might be ascribed to electrostatic interaction of positively charged chitosan with electronegative oxygen atoms of different stabilizers in slightly acidic media producing a gel with improved mechanical properties and slower drug release rate [25,26]. Doubling the amount of polymer added (from 1:2 to 1:4 drug/polymer ratio) reduced PP release, even though this reduction was statistically insignificant (*p* > 0.05).

### 3.6. Stability

The hydrophobic nature of PCL promotes poor stability of nanoparticles when exposed to water. To assess the stability of prepared formulations, they were stored at room temperature for three months and checked for particle size, zeta potential, PI, and EE% (Table 3). Although the initial results of particle size and PDI showed that F6, F8, F14, and F16 were large (>500 nm) with heterogeneous particle distribution (PDI > 0.3), these formulations did not show a significant difference in particle size and PDI after 3 months. This might be attributed to high positive values of particle surface charge (>30 mV) which in turn promoted particle-particle repulsion and mitigated aggregation. Additionally, zeta potential and encapsulation efficiency of prepared nanoparticles did not change significantly within the three months of stability investigation.

### 3.7. Thermal Analysis

**Figure 3:**
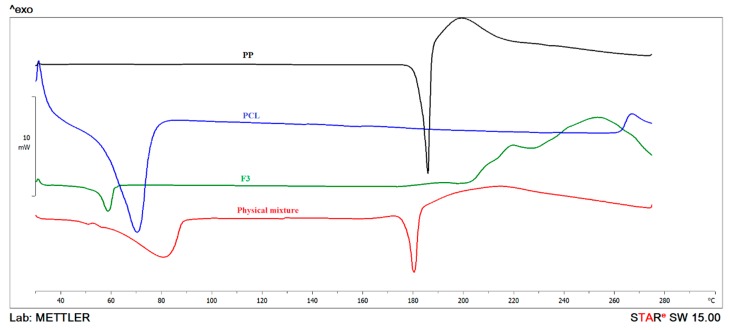
DSC thermograms of; PP, PCL, F3, and physical mixture.

To describe the crystallinity of PP and PCL individually and after formulation in polymeric nanoparticles, DSC analyses were performed (Figure 3). PP showed a sharp endothermic peak at 185.3 °C followed by a broad exothermic peak indicating drug decomposition after transition [27]. PCL showed a main endothermic peak at 69.8 °C. F3 exhibited a peak at 58.7 °C. Shifting of PCL peak into lower temperatures in the nanoparticles may be attributed to the presence of particles in the nano-range. However, the vanishing of the PP characteristic peak in the thermogram of nanoparticles suggests the existence of the crystalline drug in an amorphous state of molecular dispersion or solid solution state within the polymeric matrix [28]. In addition, the thermogram of F3 formulation showed a reduction in endothermic peak intensity. This behavior is possibly due to PCL chain disorganization occurring when pure materials are incorporated in Pluronic F127 containing system, leading to lower energy necessary to melt the polymer crystals [29]. Moreover, the presence of both peaks in the physical mixture confirmed the interaction between PP and PCL in nanoparticle formulations. It is noteworthy that F4 (chitosan-coated F3) exhibited the same thermogram of F3 suggesting possible Pluronic chitosan interaction.

### 3.8. FT-IR Analysis

**Figure 4:**
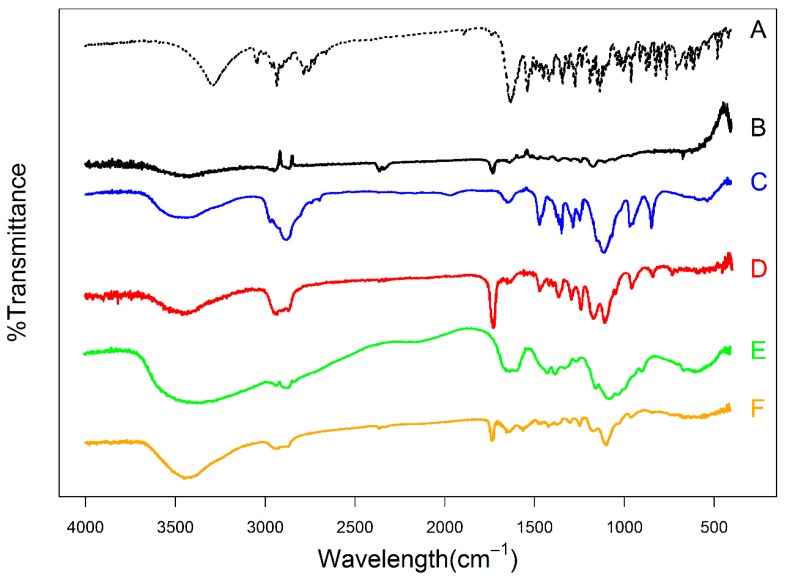
FT-IR spectra of (**A**); PP, (**B**); PCL, (**C**); Pluronic F127, (**D**); F3, (**E**); chitosan, and (**F**); F4.

Interaction (presented as bond formation) of PP and other polymeric nanoparticle components was further assessed by FT-IR. In addition to pure PP, PCL, Pluronic F127 (PF127) and chitosan, F3 and F4 FT-IR spectra are shown in Figure 4. PP spectrum showed characteristic peaks at 3294.78 cm^−1^ (represented –OH stretch), 2934.16 cm^−1^ (represented CH stretch), 1627 cm^−1^ (represented C=C stretch), 1535.06 cm^−1^ (represented C=N stretching), and 1131.05 cm^−1^ (represented C–F stretching) [30]. PCL spectrum exhibited characteristic bands at 3420.13 cm^−1^ (represented terminal hydroxyl group), 2940.6 cm^−1^, and 2867.8 cm^−1^ (represented asymmetric and symmetric aliphatic stretching respectively). In addition, the PCL spectrum showed polyester carbonyl stretching (C=O) absorption peak at 1728.5 cm^−1^ and stretching vibration bands of C–O–C bonds at 1169.9 cm^−1^ [31]. Pluronic F127 spectrum showed peaks at 2878.24 cm^−1^ and 1108.84 cm^−1^, representing C–H and C–O stretching, respectively; and an absorption band at 3437.5 cm^−1^ corresponding to O–H group stretching. After matching PP characteristic peaks with F3 peaks it is possible to say that the F3 spectrum is devoid of most PP characteristic peaks indicating successful encapsulation within polymeric nanoparticles. Moreover, the F3 spectrum contained the C–O stretching peak (1107.3 cm^−1^) of Pluronic F127 indicating surface coating by the stabilizer. Chitosan spectrum revealed characteristic bands at 1633.41 cm^−1^ representing vibration of the carbonyl group of acetylated amide and 1532.6 cm^−1^ representing stretching of the free amino group. The chitosan-coated F3 (F4) spectrum showed absence of Pluronic F127 C–O stretching peak with shifting in amide carbonyl group and amino group of chitosan confirming surface interaction between Pluronic F127 and chitosan.

### 3.9. Surface Morphology

**Figure 5:**
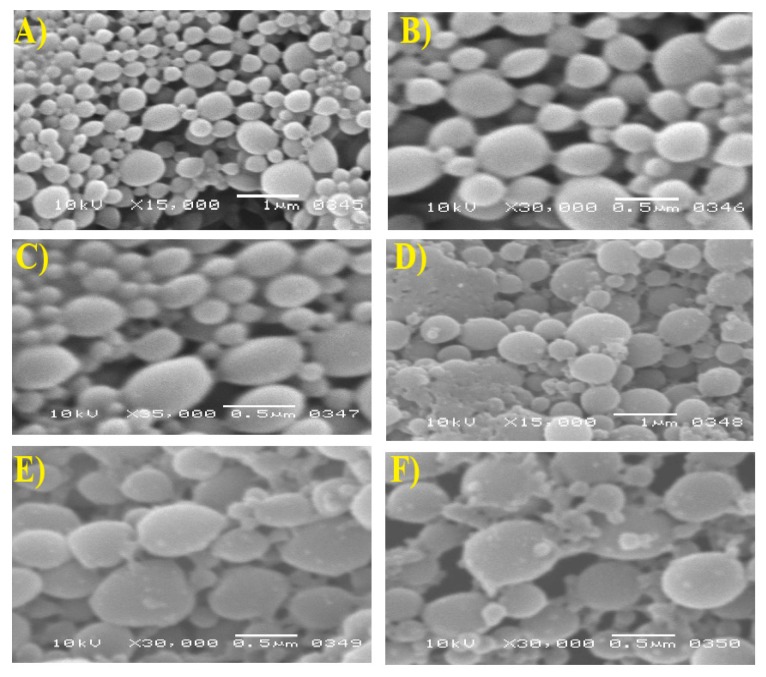
Scanning electron microscope images of (**A**) F1; (**B**) F2; (**C**) F3; (**D**) F4; (**E**) F7 and (**F**) F8.

The morphological characteristics of coated and uncoated nanoparticles were observed by SEM (Figure 5A–F). Particles were assessed for surface smoothness, discreteness, shape, and homogeneity. It was clear that the particles were of smooth surface, undetectable aggregation, and spherical shape. Particles with a smooth surface and spherical shape were reported to be of less tissue irritation than irregular and crystalline particles [32]. The particles of coated formulations appeared of a smaller size than that obtained by zeta sizer measurements which might be attributed to a hydrated layer formed on chitosan-coated formulations during measuring particle size by dynamic light scattering [33]. Dynamic light scattering reflects the hydrodynamic diameter, whereas SEM reflects the actual diameter of the dry state. Additionally, the micrographs revealed more pronounced heterogeneity in coated particle distribution compared with the corresponding uncoated particle distribution which was in good agreement with the results obtained from the zeta sizer analysis.

### 3.10. BSA Adsorption

**Figure 6:**
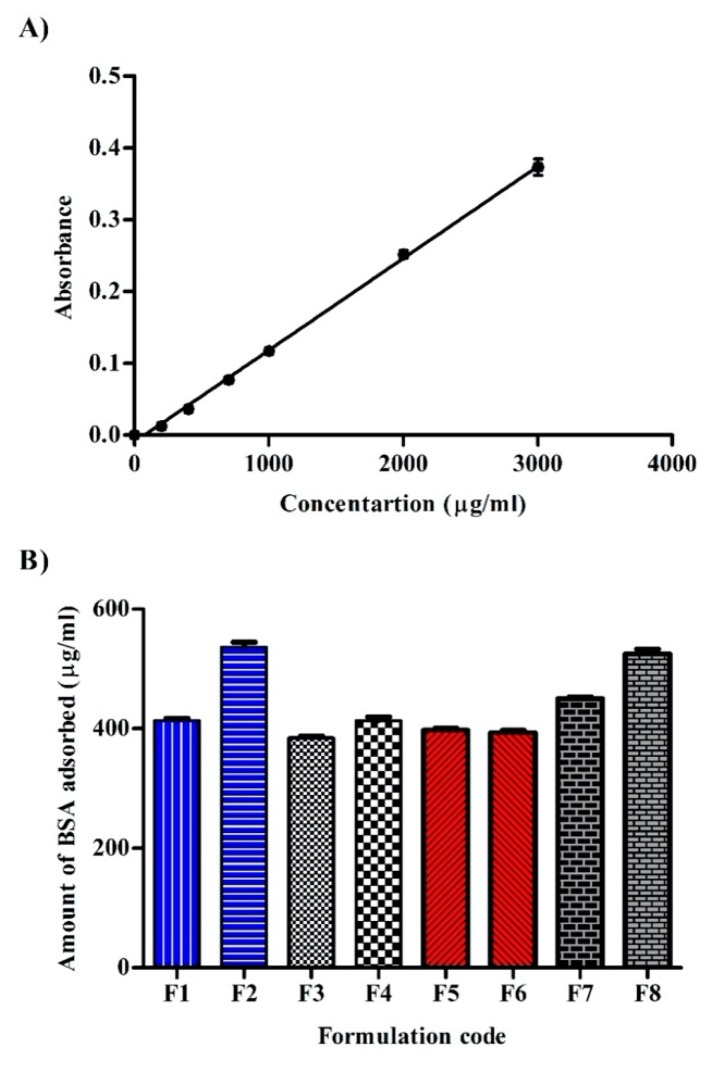
(**A**) Calibration curve of Biuret-BSA complex and (**B**) amounts of bovine serum albumin (BSA) adsorbed onto different formulations (*n* = 3).

As prepared nanoparticles are intended for parenteral administration, the amount of BSA adsorbed onto different formulations was determined by the Biuret-protein complex assay (Figure 6A,B). Protein adsorption influences subsequent phagocytosis, membrane transport and circulation half-life [34]. The degree of protein adsorption is influenced by several factors such as electrostatic interaction [35], hydrophobicity [36], surface roughness [37], and hydrogen bonding. Considering stabilizer effect, PVA based formulation (F7) exhibited the greatest BSA adsorption (450 ± 6 μg/mL) whereas Pluronic F127 based formulation (F3) exhibited the least adsorption (384.3 ± 5.5 μg/mL) among uncoated formulations. Higher BSA adsorption in the case of PVA stabilized formulations is thought to be due to lower softness of large particles [38] and hence easy protein adsorption [39]. Pluronics are triblock copolymers of polyethylene oxide (PEO)-polypropylene oxide (PPO)-polyethylene oxide (PEO), which were well established of their steric effect [40] and specifically in resisting interaction with serum components [41]. This behavior could partly explain the lower BSA adsorption in pluronic containing formulations when compared with Tween 80 and PVA. Pluronic F127 based formulation adsorbed less BSA than F68. This might be ascribed to the fact that the hydrophilic chain (PEO) responsible for the steric effect of the copolymer is more predominant in the F127 grade. The lower BSA adsorption in the case of Tween 80 based formulation compared with PVA might be related to the prevalence of negative charge on the surface of F1 which could promote repulsive force with carboxylic acid carrying BSA. On the contrary to nanoparticle stabilizers, higher PCL concentration (1:4 drug to polymer ratio; F9–F16) did not produce a significant effect on BSA adsorption when compared to corresponding formulations containing lower PCL concentration (data not shown).

Chitosan coated formulations displayed variable responses towards BSA adsorption. F2 and F8 showed significant (*p* < 0.05) higher BSA adsorption compared with F1 and F7. This could be attributed to different electrostatic interactions caused by the different pH values of the formulations. The isoelectric point (the pH at which molecule is of zero charge) of chitosan is 6.2–6.8 [42]. Below this pH, the amino group of chitosan will be protonated and carry a positive charge. In addition, the isoelectric point of BSA is 4.7–4.9 [43]. Above this pH, the carboxylic group of BSA will be dissociated and carry a negative charge. In the case of F1 (pH 6.4 ± 0.41) and F7 (pH 6.1 ± 0.92), chitosan amino groups are less protonated compared with F2 (pH 5.6 ± 0.73) and F8 (pH 5.4 ± 0.58), thus attract less of BSA dissociated carboxylic groups. Interestingly, chitosan coating of Pluronic stabilized formulations did not produce significant (*p* > 0.05) BSA adsorption which might be ascribed to electrostatic interaction between protonated chitosan and electronegative oxygen atom in the POE part of Pluronic which in turn restricted the interaction of chitosan with BSA.

### 3.11. Cell Viability

**Figure 7:**
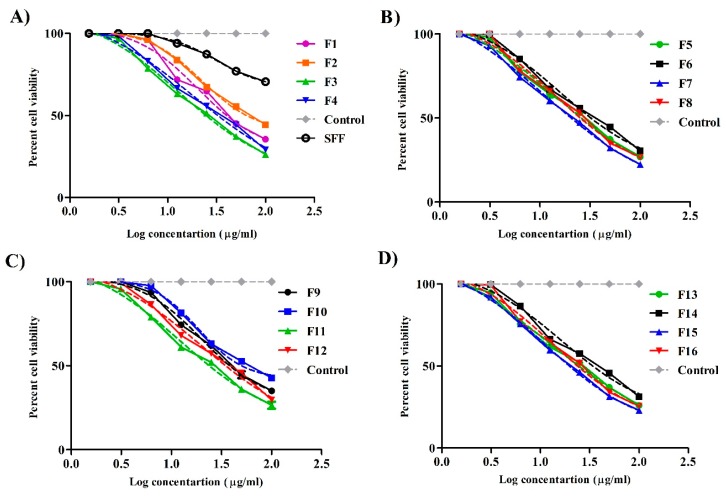
Percentage of cell viability of WI-38 cell line incubated with culture media (control) and different concentrations of stabilizer free formulation (SFF) and all other investigated formulations ((**A**), for F1–F4; (**B**), for F5–F8; (**C**), for F9–F12; (**D**), for F13–F16) after 48 h by MTT assay (*n* = 3). Continuous lines represent the actual curves of the original values while dashed lines represent curves after a non-linear fit of data of corresponding colors.

The percentage of viable WI-38 cells after 48 h determined via MTT assay is depicted in Figure 7A–D. The 48 h period is long enough to check and predict long term cellular toxicity. Results showed that all formulations produced cell toxicity in a concentration-dependent manner. The higher the nanoparticle concentration, the more cytotoxic they were. SFF showed considerable cell viability percentage (70.6 ± 2.1% of cells were viable after incubation with the highest concentration) and higher IC50 (31.6 ± 1.7 μg) which were significantly (*p* < 0.05) higher than that of stabilizer containing formulations. This result confirms the safety of PCL on the molecular level [44] even in high molecular weights.

Stabilizer containing formulations in the higher concentration (100 μg/mL) caused the death of approximately 70% of the viable cells depending on formulation constituents, although all formulations at the lowest concentration (1.56 μg/mL) did not exhibit any cell death. IC50 values of such formulations were also significantly decreased when compared with SFF. IC50 values (Table 4) were 20.65 ± 0.7 μg, 13.27 ± 0.4 μg, 13.91 ± 0.5 μg, 11.56 ± 0.08 μg, 20 ± 1.4 μg, 12.94 ± 0.7 μg, 11.99 ± 0.07 μg, 11.27 ± 0.09 μg for F1, F3, F5, F7, F9, F11, F13, and F15 respectively. These findings simply imply that stabilizer concentration represents a crucial factor affecting cellular safety which is in good agreement with the literature data. Grabowski et al. reported that the toxicity of polymeric nanoparticles could be mediated by surface coating with different stabilizers [45]. Abriata et al. [15] showed that Pluronic F127 stabilized PCL nanoparticles did not exhibit any cytotoxic effect on the LLC-MK2 cell line at the highest concentration used (120 μM). The investigators used lower molecular weight PCL (14,000 Da) and different cell lines (LLC-MK2 cells), which might explain the different findings relative to our study.

All chitosan-coated formulations exhibited less cytotoxic effect than the corresponding uncoated ones at all concentrations. IC50 values were 22.5 ± 2.8 μg, 18.37 ± 1.4 μg, 16.23 ± 0.9 μg, 14.49 ± 1.1 μg, 18.66 ± 0.7 μg, 20.48 ± 1.3 μg, 16.68 ± 0.8 μg, 13.65 ± 0.08 μg for F2, F4, F6, F8, F10, F12, F14, and F16 respectively. Generally, cationic polymers interact with the anionic constituent of glycoprotein (sialic acid) and produce cell damage. However, chitosan was reported to be less toxic than other cationic polymers as polyethyleneimine [46]. Chitosan can mediate cellular damage if present in a charged form. In our study, the formulations were diluted with culture media of pH 7.4, the pH at which chitosan is mostly uncharged [47] which in turn masked its toxicity.

### 3.12. Cytokine Secretions

**Figure 8:**
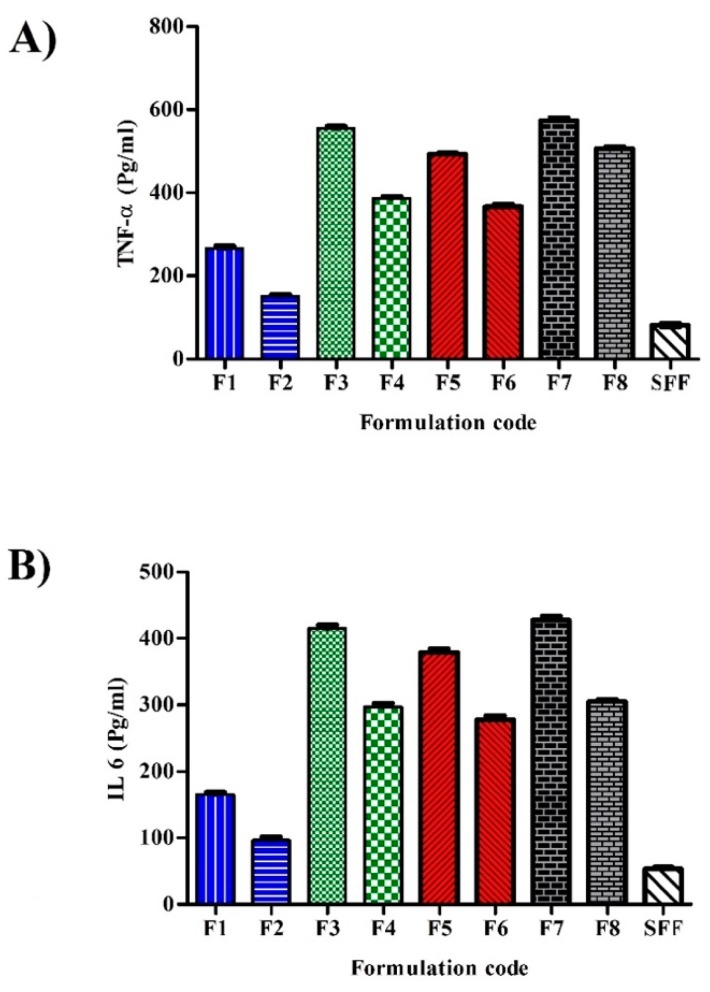
Secretions of (**A**) TNF-α and (**B**) IL-6 of SFF and different stabilizers containing formulations after incubation with 50 μg/mL after 24 h (*n* = 3).

The cellular inflammatory response was investigated by the determination of TNF-α and IL-6 (Figure 8A,B respectively). Although SFF showed a slight release of both TNF-α and IL-6, it still exhibited significantly lower levels than stabilizers containing formulations confirming the crucial role of stabilizers in triggering inflammatory responses. Grabowski et al. suggested that nanoparticles could provoke stabilizer cellular internalization which could not occur if used in a free form [45]. The formulation F1 (stabilized by Tween 80) showed the lowest TNF-α and IL-6 secretions (266 ± 3.7 and 156.3 ± 2.8 Pcg/mL) among all uncoated formulations. The extent of secretion was ranked as: F7 > F3 > F5 > F1. The results of IL-6 are in good accordance with Schöler et al. who studied the effect of different stabilizers on cytotoxicity and cytokine production of solid lipid nanoparticles [48]. The authors found that Tween 80 produced less IL-6 than Pluronics. Chitosan coated formulations showed significantly lower TNF-α and IL-6 secretions when compared to the corresponding uncoated formulations confirming the good biocompatibility and minimum cellular interaction of chitosan. Unlike stabilizer and chitosan coating, PCL concentration could not be associated with TNF-α and IL-6 secretions where no statistical significance was observed when the higher polymer drug ratio (4:1) was used.

### 3.13. Oxidative Stress

**Figure 9:**
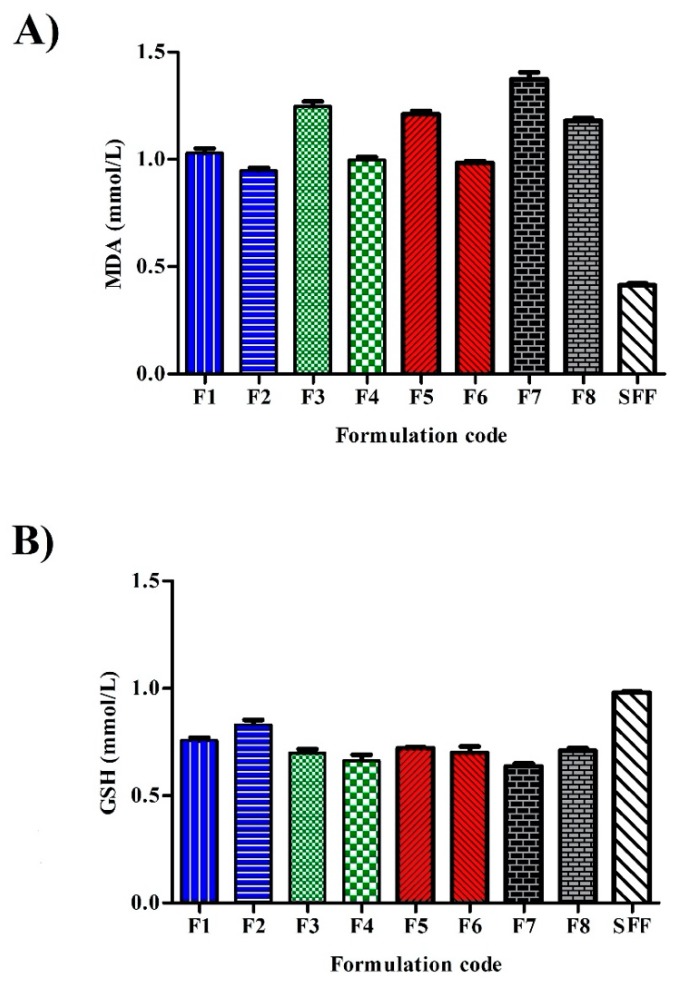
Intracellular production of (**A**) malondialdehyde (MDA) and (**B**) reduced glutathione (GSH) after exposure to SFF and different stabilizers containing formulations after incubation with 50 μg/mL after 24 h (*n* = 3).

Oxidative stress was investigated by the determination of MDA and GSH (Figure 9A,B, respectively). MDA is the end product of lipid peroxidation of which high level designates tissue damage and failure of anti-oxidant mechanisms [49]. GSH is the major non-protein thiol in living organisms that can remove free radical species and preserving membrane integrity. Significantly, lower MDA (0.41 ± 0.03 mmoL/L) and higher GSH (0.98 ± 0.05 mmoL/L) levels were seen in SFF incubated cells when compared to surfactant stabilized formulations, confirming the critical role of surfactants in cell survival. The results of oxidative stress corroborated with previous results of cell viability and inflammatory responses and evidenced the ability of chitosan-coated PCL nanoparticles to minimize stabilizer cytotoxic effects and contribute to their safety index.

## 4. Conclusions

PP loaded PCL nanoparticles can be successfully prepared by the nanoprecipitation technique. Surfactants can produce spherical nanosized particles with low polydispersity index, negative surface charge, and high encapsulation ability. Chitosan coating increases particle size and shifts surface charge to positive values. PP is slowly released from the nanoparticles in a biphasic mode. The highest sustainability is achievable by coating Pluronic stabilized nanoparticles with chitosan. Among tested stabilizers, Pluronic F127 adsorb the least amount of protein and Tween 80 triggers the least influence on cytokine secretions and oxidative stress markers. Cell viability depends on stabilizer concentration rather than the stabilizer type. Chitosan-coating minimizes stabilizer induced cytotoxicity, cytokine secretion, and oxidative stress response, although it augments nanoparticle protein adsorption capacity. The effect of chitosan on protein adsorption is neutralized when coupled with Pluronics stabilizer. Our results indicate that stabilizer type/concentration and chitosan coating play critical roles in developing safe and effective long-acting PCL nanoparticles intended for parenteral drug delivery. Based on the analysis provided above, the formulations F2 and F4 (chitosan-coated formulations containing Tween 80 and Pluronic F127 as stabilizers, respectively) are warranted a future in vivo study to delineate its safety and efficacy profiles.

## Figures and Tables

**Table 1:** Compositions of different paliperidone (PP)-loaded polycaprolactone (PCL) nanoparticle batches.

Code	Surfactant (0.5%)	Drug/Polymer Ratio	Chitosan Coating
F1	Tween 80	1/2	Uncoated
F2	Tween 80	1/2	Coated
F3	Pluronic F127	1/2	Uncoated
F4	Pluronic F127	1/2	Coated
F5	Pluronic F68	1/2	Uncoated
F6	Pluronic F68	1/2	Coated
F7	PVA	1/2	Uncoated
F8	PVA	1/2	Coated
F9	Tween 80	1/4	Uncoated
F10	Tween 80	1/4	Coated
F11	Pluronic F127	1/4	Uncoated
F12	Pluronic F127	1/4	Coated
F13	Pluronic F68	1/4	Uncoated
F14	Pluronic F68	1/4	Coated
F15	PVA	1/4	Uncoated
F16	PVA	1/4	Coated

**Table 2:** Particle size, PDI, zeta potential, yield percent and EE% of PP loaded nanoparticles (*n* = 3, ± SD).

Code	Particle Size (nm)	PDI	Zeta Potential (mV)	Yield%	pH	EE%
F1	169.1 ± 5.4	0.116 ± 0.051	−9.48 ± 0.56	68.2 ± 3.1	6.4 ± 0.41	58.6 ± 3.4
F2	246.9 ± 3.5	0.181 ± 0.084	31.73 ± 0.48	62.3 ± 2.4	5.6 ± 0.73	58.3 ± 2.6
F3	160.8 ± 3.7	0.073 ± 0.058	−6.67 ± 1.04	79.4 ± 5.8	6.2 ± 0.39	64.8 ± 5.8
F4	388.6 ± 10.6	0.351 ± 0.122	36.71 ± 1.26	76.9 ± 1.8	5.7 ± 0.52	62.3 ± 7.6
F5	152.4 ± 4.7	0.076 ± 0.058	−12.9 ± 2.83	72.6 ± 7.3	6.4 ± 0.40	63.6 ± 5.8
F6	451.2 ± 36.3	0.461 ± 0.073	32.53 ± 1.20	70.4 ± 8.1	5.3 ± 0.17	59.7 ± 1.5
F7	360.3 ± 28.5	0.654 ± 0.156	−4.57 ± 0.63	38.6 ± 3.7	6.1 ± 0.92	51.6 ± 3.3
F8	415.3 ± 20.8	0.439 ± 0.100	31.83 ± 2.51	32.1 ± 6.9	5.4 ± 0.58	51.8 ± 6.2
F9	166.4 ± 1.3	0.100 ± 0.031	−12.91 ± 0.59	64.7 ± 5.3	6.6 ± 0.28	64.2 ± 1.7
F10	361.2 ± 17.1	0.345 ± 0.029	27.37 ± 0.64	61.9 ± 1.9	5.7 ± 0.36	65.1 ± 2.3
F11	180.5 ± 4.9	0.405 ± 0.030	−8.03 ± 0.587	75.2 ± 6.3	6.3 ± 0.12	70.6 ± 6.8
F12	257.4 ± 8.3	0.313 ± 0.069	34.13 ± 0.25	72.6 ± 5.9	5.7 ± 0.73	69.5 ± 2.9
F13	164.8 ± 0.5	0.094 ± 0.040	−15.53 ± 0.28	66.3 ± 4.8	6.6 ± 0.18	66.3 ± 3.6
F14	453.5 ± 17.4	0.464 ± 0.005	37.83 ± 0.46	61.4 ± 3.9	5.2 ± 0.24	65.1 ± 2.8
F15	368.5 ± 18.5	0.397 ± 0.058	−5.41 ± 0.82	34.5 ± 7.4	6.3 ± 0.46	56.4 ± 9.2
F16	616.4 ± 30.6	0.468 ± 0.019	31.37 ± 0.89	32.8 ± 2.6	5.5 ± 0.25	52.3 ± 8.4

**Table 3:** Particle size, PDI, zeta potential, and EE% of PP loaded nanoparticles within 3 months (*n* = 3, ±SD).

Code	1st Month				2nd Month				3rd Month			
	Particle Size (nm)	PDI	Zeta Potential (mV)	EE%	Particle Size (nm)	PDI	Zeta Potential (mV)	EE%	Particle Size (nm)	PDI	Zeta Potential (mV)	EE%
F1	179.3 ± 2.4	0.12 ± 0.043	−7.6 ± 0.41	59.2 ± 7.3	203.3 ± 8.3	0.140 ± 0.090	−8.37 ± 0.22	57.1 ± 1.6	213.1 ± 11.2	0.151 ± 0.076	−8.87 ± 0.59	58.3 ± 3.6
F2	256.7 ± 6.1	0.192 ± 0.057	35.39 ± 1.8	57.1 ± 3.4	277.7 ± 9.7	0.163 ± 0.031	39.60 ± 2.17	58.1 ± 6.1	284.3 ± 9.5	0.176 ± 0.043	39.63 ± 1.84	57.6 ± 3.4
F3	170.4 ± 5.6	0.09 ± 0.017	−6.12 ± 0.63	63.5 ± 2.6	180.4 ± 1.8	0.155 ± 0.026	−5.27 ± 1.63	61.4 ± 4.3	182.9 ± 1.6	0.111 ± 0.016	−5.91 ± 0.38	62.2 ± 1.5
F4	358.5 ± 9.7	0.376 ± 0.094	38.43 ± 4.9	63.8 ± 4.9	369.5 ± 7.8	0.303 ± 0.081	42.37 ± 2.91	63.1 ± 8.3	385.7 ± 6.4	0.344 ± 0.086	38.74 ± 1.69	64.2 ± 1.9
F5	165.3 ± 4.8	0.093 ± 0.036	−13.1 ± 3.4	61.4 ± 1.9	168.1 ± 3.2	0.167 ± 0.055	−14.3 ± 1.07	62.8 ± 4.6	175.9 ± 3.5	0.135 ± 0.033	−1122 ± 0.77	61.7 ± 8.2
F6	571.8 ± 11.6	0.458 ± 0.029	30.23 ± 6.2	57.1 ± 4.6	586.6 ± 29.7	0.428 ± 0.017	27.53 ± 1.34	56.5 ± 7.3	587.4 ± 28.4	0.430 ± 0.075	32.06 ± 2.47	54.9 ± 5.3
F7	369.2 ± 13.2	0.652 ± 0.240	−5.12 ± 0.09	52.3 ± 1.9	373.4 ± 31.2	0.627 ± 0.093	−6.45 ± 1.19	50.1 ± 3.1	429.7 ± 33.2	0.487 ± 0.078	−5.81 ± 1.62	51.0 ± 2.8
F8	525.7 ± 9.8	0.452 ± 0.072	30.34 ± 6.5	51.8 ± 6.2	559.3 ± 26.3	0.499 ± 0.022	29.97 ± 1.70	52.7 ± 4.5	564.3 ± 24.3	0.596 ± 0.018	36.06 ± 3.72	50.9 ± 6.5
F9	179.3 ± 6.5	0.115 ± 0.028	−15.22 ± 1.6	63.9 ± 4.1	193.9 ± 8.7	0.118 ± 0.032	−17.1 ± 1.48	62.3 ± 9.3	197.4 ± 14.1	0.146 ± 0.063	−14.43 ± 2.60	61.6 ± 2.4
F10	370.3 ± 8.3	0.367 ± 0.083	27.4 ± 2.4	62.1 ± 4.6	379.0 ± 17.3	0.388 ± 0.046	27.83 ± 1.64	61.6 ± 8.6	382.4 ± 15.3	0.391 ± 0.047	27.95 ± 1.35	61.0 ± 7.3
F11	230.1 ± 3.9	0.398 ± 0.047	−7.3 ± 0.64	71.3 ± 2.9	250.1 ± 10.2	0.416 ± 0.027	−6.75 ± 1.02	70.9 ± 6.8	255.2 ± 14.6	0.499 ± 0.0477	7.84 ± 0.07	70.8 ± 2.1
F12	269.6 ± 1.9	0.288 ± 0.071	36.84 ± 2.1	68.3 ± 7.3	300.4 ± 5.7	0.238 ± 0.012	39.71 ± 6.60	67.4 ± 1.7	314.7 ± 6.7	0.251 ± 0.015	35.67 ± 1.86	66.7 ± 5.4
F13	181.9 ± 7.5	0.113 ± 0.085	−14.6 ± 0.99	65.1 ± 5.6	219.3 ± 2.4	0.230 ± 0.0473	−13.3 ± 1.39	66.2 ± 2.6	222.6 ± 6.2	0.205 ± 0.040	−12.29 ± 2.04	64.3 ± 2.7
F14	493.1 ± 10.6	0.415 ± 0.032	37. 3 ± 4.6	62.9 ± 1.9	496.1 ± 13.6	0.409 ± 0.026	38.70 ± 1.75	60.1 ± 6.9	496.3 ± 22.9	0.422 ± 0.063	39.67 ± 1.34	61.3 ± 8.1
F15	273.8 ± 2.4	0.393 ± 0.022	−5.37 ± 0.17	52.1 ± 7.3	286.3 ± 5.1	0.391 ± 0.024	−5.53 ± 1.78	53.1 ± 4.4	297.3 ± 4.8	0.298 ± 0.086	−4.71 ± 0.89	52.6 ± 6.2
F16	626.4 ± 13.1	0.455 ± 0.056	33.46 ± 3.5	53.5 ± 1.6	673.3 ± 38.2	0.454 ± 0.044	38.37 ± 5.41	52.2 ± 5.8	683.7 ± 36.3	0.395 ± 0.026	34.92 ± 2.40	51.8 ± 3.3

**Table 4:** IC50 of different PP loaded PCL nanoparticle batches after incubation with WI-38 cells for 48 h.

Code	IC50 (μg ± SD)	Code	IC50 (μg ± SD)
F1	20.65 ± 0.7	F9	20 ± 1.4
F2	22.5 ± 2.8	F10	18.66 ± 0.7
F3	13.27 ± 0.4	F11	12.94 ± 0.7
F4	18.37 ± 1.4	F12	20.48 ± 1.3
F5	13.91 ± 0.5	F13	11.99 ± 0.07
F6	16.23 ± 0.9	F14	16.68 ± 0.8
F7	11.56 ± 0.08	F15	11.27 ± 0.09
F8	14.49 ± 1.1	F16	13.65 ± 0.08
